# Demand forecasting and inventory optimization of distribution equipment: A fusion model based on genetic algorithm and machine learning

**DOI:** 10.1371/journal.pone.0336026

**Published:** 2025-11-18

**Authors:** Qingbo Tu, Hongyang Zhang, Weiwei Li, Jing Duan, Chao Kong

**Affiliations:** 1 Economic and Technological Research Institute, State Grid Shandong Electric Power Company, Jinan, China; 2 State Grid Economic and Technical Research Institute Co., Ltd., Beijing, China; King Fahd University of Petroleum & Minerals, SAUDI ARABIA

## Abstract

To improve the intelligent and refined management level of power distribution systems in equipment operation and maintenance as well as emergency support, this work proposes an integrated “prediction-optimization” model that combines genetic algorithm (GA) with machine learning methods. This method uses GA to intelligently screen key features and optimize model parameters. It dynamically integrates the prediction link with inventory decisions, alleviating the problem of multi-objective coupling imbalance in traditional fragmented optimization. Compared with a single machine learning or heuristic algorithm, this model significantly reduces the unit prediction error under load fluctuations and extreme weather scenarios. Verification of model performance based on The European Network of Transmission System Operators for Electricity (ENTSO-E) dataset shows that the model achieves good results in the prediction stage. For example, in load time series data, the mean absolute percentage error is 3.41%, and the coefficient of determination reaches 0.942. In the inventory optimization stage, the model reduces the average inventory level to 42.63, controls the total cost per unit equipment at 92.37, and lowers the redundant inventory ratio to 9.42%. Its comprehensive performance is better than that of Temporal Fusion Transformer (TFT) and Neural Basis Expansion Analysis for Time Series Forecasting (N-BEATS). This work provides theoretical models and empirical support for research in the field of typical equipment prediction and inventory optimization in intelligent power distribution systems, and has certain practical value and promotion significance.

## Introduction

With the rapid advancement of new power systems and intelligent distribution networks, distribution systems are playing an increasingly vital role in ensuring power quality, supporting renewable energy integration, and enhancing power supply reliability [1–4]. Typical equipment, such as circuit breakers, transformers, switches, and cables, serves as essential infrastructure in distribution systems. The strategies for their procurement and reserve directly impact the safety, cost-effectiveness, and emergency responsiveness of system operations [5]. However, due to multiple factors such as load fluctuations, climate change, equipment aging, and policy adjustments, the demand for typical equipment exhibits significant dynamism and uncertainty. This creates considerable challenges in formulating procurement plans and managing inventory reserves. Traditional forecasting methods are primarily based on linear regression or heuristic rules, and they struggle to capture complex time-series variations and nonlinear dynamic relationships with sufficient accuracy [6]. Meanwhile, in terms of procurement and reserve strategies, many distribution companies still rely on experience-based approaches, resulting in low forecasting accuracy, high inventory costs, and inefficient resource allocation. For example, due to unreasonable inventory allocation, a provincial power grid enterprise has an equipment idle rate exceeding 15%, with annual capital occupation exceeding 230 million yuan. In another city, due to the lack of accurate demand forecasting, key ring main unit spare parts are out of stock during emergency repair work after a typhoon. This results in a 7-hour delay in fault recovery, expanding the scope of power outage impact. Especially in the context of large-scale integration of clean energy and accelerated grid digitalization, traditional models can no longer meet the growing demand for efficient, accurate, and intelligent power equipment management. In recent years, machine learning techniques have achieved notable progress in power systems, particularly demonstrating strong modeling capabilities in areas such as load forecasting, fault diagnosis, and condition assessment [7]. Meanwhile, the GA, as a global optimization approach, has shown robust adaptability and reliability in multi-parameter tuning, feature selection, and solving complex optimization problems. This work adopts the combination of GA and machine learning mainly for the following reasons: In terms of computational efficiency, compared with simulated annealing GA (such as the chaotic time-varying model applied to wind power inspection paths by Kou, Wang [8]) which need to handle complex chaotic mechanisms, the standard GA converges faster in feature selection and parameter optimization. Regarding the multi-objective adaptability, unlike the multi-adaptive firefly swarm GA proposed by Majhi, Kabat [9], which focused on dynamic scheduling of cloud resources, the proposed model needs to better balance the multi-objective coupling relationship between prediction accuracy and inventory cost. For the fundamentality of time-series modeling, the demand forecasting task for power distribution equipment needs to include time-series features such as load and meteorology. Machine learning models have a stronger ability to capture time-series nonlinear relationships than pure heuristic algorithms. Therefore, combining the genetic algorithm with machine learning models to develop demand forecasting frameworks and extending this integration to equipment procurement and reserve optimization holds promise for enhancing prediction accuracy and inventory management. It enables an intelligent upgrade of typical equipment operation and maintenance in distribution systems [10]. Based on this, the objectives of this work are as follows:

(1) Utilizing load time-series data, equipment operation and maintenance information, and meteorological data from the European Network of Transmission System Operators for Electricity (ENTSO-E) database, this work performs data modeling through a sliding window mechanism and feature engineering methods. A genetic algorithm is employed to jointly optimize input features and model parameters, aiming to improve the accuracy and stability of equipment demand forecasting.
(2) Based on the forecasting results, this work aims to construct a multi-objective optimization model that incorporates procurement costs, inventory holding costs, and stockout penalty costs. Constraints such as inventory balance, safety stock levels, and supply capacity are set. A global solution is derived using a genetic algorithm to optimize the inventory structure and resource allocation efficiency.
(3) Based on model experiment results and inventory optimization characteristics, this work seeks to propose optimization recommendations for equipment procurement and inventory management suited to power grid enterprises. It also intends to assess the feasibility of deploying the model in actual system scheduling and edge computing platforms to provide technical support for intelligent decision-making in distribution system operations and maintenance.

The main innovative contributions of this work include three aspects: 1) A closed-loop prediction-optimization mechanism is constructed. By dynamically linking equipment demand forecasting and inventory decision-making through GA, it alleviates the problem of response lag caused by traditional fragmented management. 2) A feature-parameter collaborative optimization method is proposed. GA is used to simultaneously screen key features and optimize model parameters, resulting in a reduction in prediction errors compared with traditional methods in scenarios of sudden load changes. 3) A multi-objective inventory model is built, which comprehensively optimizes procurement costs, stockout risks, and inventory efficiency. It reduces the redundancy rate while ensuring that the number of stockouts is lower than the industry average. In summary, this work leverages real-world power system data from ENTSO-E and integrates the global optimization capability of genetic algorithms with the predictive modeling strengths of machine learning. The work develops an integrated model for forecasting typical equipment demand and optimizing procurement reserves in distribution systems. The goal is to offer power enterprises more scientific and efficient decision-making support for equipment operation and procurement, thereby contributing to the high-quality development of intelligent distribution systems.

### Literature review

With the ongoing development of smart grids and distribution systems, equipment forecasting and inventory optimization have become important research directions in power system operation and maintenance management. Schmidl S [11] found that the demand for typical equipment in distribution systems was closely related to load fluctuations, equipment lifecycle, and environmental factors. By establishing time-series models, basic trend forecasting could be achieved. Their study provides an initial data modeling approach for demand analysis, but there are limitations in multi-factor nonlinear modeling. Barrientos-Torres, Martinez-Ríos [12] combined the Autoregressive Integrated Moving Average (ARIMA) model with multiple regression methods to predict the replacement cycles of key equipment such as transformers. Such a study is representative in terms of historical data modeling but lacks high precision in handling abnormal fluctuations and high-frequency variations, making it difficult to meet forecasting needs under dynamic operating conditions. Papazoglou and Biskas [13] found that applying the genetic algorithm to feature selection and hyperparameter optimization in machine learning models significantly improved the model’s prediction accuracy and stability. Their research focused on load forecasting and validated the effectiveness of the integrated optimization algorithm, but did not explore its application value at the equipment level in depth. Lotfi [14] further proposed a neural network model optimized by genetic algorithms and applied it to wind power output forecasting, achieving high accuracy. However, the model has a high computational complexity, posing challenges for practical system deployment, and its applicability in typical equipment forecasting still requires further validation. Pasupuleti, Thuraka [15] developed an optimal inventory model for transmission and distribution equipment, and incorporated multi-objective programming methods to optimize inventory costs and stockout risks simultaneously. While it has reference value for strategy development, it does not adequately consider the linkage between equipment forecasting and inventory management. Tanvir Rahman, Jafrin [16] proposed the introduction of a risk assessment mechanism to construct a safety stock model suitable for multi-period rolling procurement scenarios. Their study focused on the dynamic adjustment of inventory security levels but weakly handled the sensitivity to upstream equipment forecasting errors, which may lead to procurement deviations. Marković, Bossart [17] systematically explored the research progress of machine learning in power distribution systems, pointing out the core role of intelligent algorithms in equipment condition monitoring, demand forecasting, and integration of distributed energy. Their research provided methodological support for the construction of feature engineering in the equipment prediction model of this work. Wu, Yuan [18] proposed a distribution network carrying capacity analysis method based on spatiotemporal deep learning. Its innovation lied in coupling and modeling spatial topological relationships with temporal dynamic characteristics. Although the research focused on the field of power grid planning, its idea of spatiotemporal feature fusion was inspiring. This idea was particularly relevant for the cross-dimensional correlation modeling of environmental meteorological data and equipment operation and maintenance data in this work. It indirectly verified the applicability of GA in multi-variable optimization.

It can be observed that most studies adopt linear or single machine learning models, lacking systematic integration of feature engineering and model parameter optimization. Additionally, equipment demand forecasting and inventory optimization are often treated as separate areas of research, without forming an integrated “forecasting—decision-making—procurement” mechanism. Therefore, this work constructs an equipment forecasting model that combines the genetic algorithm with machine learning. The model leverages the advantages of global optimization and nonlinear modeling to enhance forecasting accuracy. Additionally, an integrated framework for forecasting and procurement reserves is proposed to link demand forecasting results with inventory strategies to achieve closed-loop management.

### Research methodology

#### Equipment demand forecasting and procurement reserve optimization framework.

**Fig 1:**
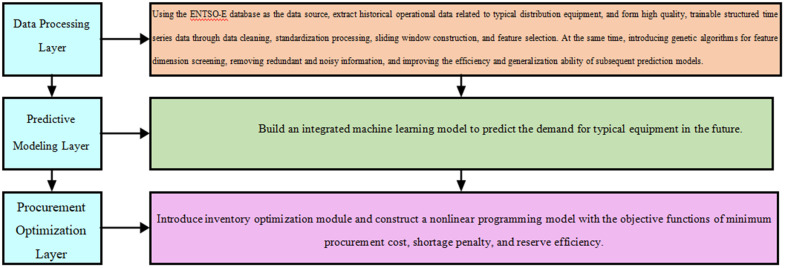
Equipment demand forecasting and procurement reserve optimization framework.

To address the issues of significant demand fluctuations, delayed procurement responses, and unreasonable inventory configurations for typical equipment in distribution systems, this work designs an integrated “forecasting—optimization—decision-making” model framework that combines the genetic algorithm with machine learning. The framework aims to achieve high-accuracy equipment demand forecasting and scientifically rational procurement reserve management. The framework consists of three core layers: the data processing layer, the forecasting modeling layer, and the procurement optimization layer. Fig 1 displays the overall process:

The data processing layer of the model uses the ENTSO-E database as the data source. It extracts historical operational data related to typical distribution equipment, including load curves, equipment replacement records, voltage fluctuations, and meteorological conditions. Through data cleaning, normalization, sliding window construction, and feature selection, high-quality structured time-series data are generated for training purposes [19]. Meanwhile, a genetic algorithm is introduced for feature dimension selection to remove redundant and noisy information, and enhance the efficiency and generalization ability of subsequent forecasting models. The core of the forecasting modeling layer is to construct an ensemble machine learning model to forecast the future demand for typical equipment over a given period [20]. This work selects support vector regression, random forests, and long short-term memory networks as base models. A genetic algorithm is used to globally optimize their hyperparameters and input feature combinations to obtain the optimal predictive model structure. The output of this layer is the predicted demand distribution for various types of equipment within the future T time window, which guides subsequent inventory configuration. Based on the equipment demand forecast results, the model incorporates an inventory optimization module. Moreover, a nonlinear programming model with the objective function of minimizing procurement costs, stockout penalties, and reserve efficiency is constructed [21]. This model takes forecasted demand as an input variable. Then, combining multi-dimensional constraints such as historical procurement cycles, equipment delivery time, and safety stock requirements, it uses a genetic algorithm to solve for the optimal inventory configuration. The output is the dynamic procurement quantity and reserve plan for various types of typical equipment. The greatest advantage of this framework is the realization of a closed-loop linkage between data-driven forecasting and inventory optimization. It can simultaneously balance forecasting accuracy and operational costs, and improve the intelligence and scientific decision-making in the operation and maintenance management of distribution systems.

### Data preprocessing and feature engineering

The quality of data directly determines the performance of the forecasting model. For the operational data and load data of typical equipment in the distribution system from the ENTSO-E database, this work conducts systematic data preprocessing and feature engineering before modeling. This aims to ensure that the model input is representative, accurate, and interpretable. The process mainly includes four steps: data cleaning, feature construction, sliding window modeling, and feature selection.

The raw data contains some missing values, duplicates, and outliers. The following strategies are used to handle these issues:

(1) Missing Value Imputation: For continuous variables, linear interpolation and mean filling are used. For categorical variables, the nearest neighbor imputation method is applied.
(2) Outlier Removal: Using boxplot methods and the 3σ principle, outliers in key indicators such as load, voltage, and current are identified and extreme deviation values are removed [22–24].
(3) Data Unification and Format Conversion: Since different data sources in the ENTSO-E database have varying frequencies, it is necessary to unify the time granularity. An hourly time resolution is adopted as the basis for modeling in this study.

To comprehensively reflect the dynamic characteristics affecting equipment demand, this work constructs multi-dimensional feature variables based on the original indicators, including:

(1) Time-related Features: Time of day, daily cycle, weekdays/weekends, month, and season.
(2) Load Dynamics Features: Current load, past k-period load averages, maximum, minimum, and standard deviation.
(3) External Environmental Features: Meteorological variables such as temperature, humidity, and wind speed.
(4) Equipment Status Features: Operating time, historical fault frequency, and average maintenance cycle.

Additionally, a sliding window mechanism is used to structure the time series, converting the continuous time series into a supervised learning task, making it suitable for machine learning model training.

To avoid an excessive input dimension that would increase computational complexity and reduce prediction performance due to redundant features, a genetic algorithm is introduced to search for the optimal feature subset combination:

(1) Encoding Method: Binary encoding is used, where each bit represents whether a feature is selected or not [25].
(2) Fitness Function: The error metrics of the prediction model on the validation set are used to evaluate the fitness.
(3) Evolutionary Mechanism: Parameters such as crossover rate, mutation rate, and population size are set, and the optimal feature combination is obtained through multiple generations of evolution.
(4) Result Output: The final set of efficient feature subsets, which contribute the most to equipment demand forecasting, is selected as the model input.

Since different features have different dimensions and scales, Min-Max normalization is applied to scale all numerical variables to the [0,1] range to avoid model bias toward variables with higher values. This also enhances the stability of model training and convergence speed [26,27].

Through the above data preprocessing and feature engineering process, this work builds a clear, information-rich, and predictive data input foundation, providing strong support for subsequent forecasting modeling and inventory optimization.

#### Design of procurement and reserve optimization model.

After completing the demand forecasting for typical equipment, the key step in improving the resource allocation efficiency and operation and maintenance response capabilities of the distribution system is to convert the forecast results into scientifically rational procurement and reserve decisions. To this end, this work constructs a multi-constraint nonlinear inventory optimization model intending to minimize the total equipment procurement cost, while considering stockout risks and inventory efficiency. A genetic algorithm is used to solve and optimize the model.

The core objective of the model is to optimize the inventory structure and procurement batch while minimizing the following integrated costs, ensuring the reliable supply of equipment:

(1) Procurement Cost: The cost of procuring each type of equipment, calculated by multiplying the unit price by the procurement quantity for a given time period.
(2) Inventory Holding Cost: The unit time cost of storing equipment in inventory.
(3) Stockout Penalty Cost: The additional costs incurred due to operational delays, activation of backup mechanisms, or other issues caused by equipment shortages.

Based on the power equipment inventory management standard model [28], the objective function is designed as follows:


$$minC=\sum_{i=\text{1}}^{n}\;\left(c_{i}^{p}\cdot Q_{i}+c_{i}^{h}\cdot I_{i}+c_{i}^{s}\cdot S_{i}\right)$$ (1)

$Q_{i}$ is the procurement quantity of the i-th type of equipment, $I_{i}$ is the inventory at the end of the period for the i-th type of equipment, and $S_{i}$ is the expected stockout quantity for the i-th type of equipment. $c_{i}^{p}$, $c_{i}^{h}$, and $c_{i}^{s}$ correspond to the unit procurement cost, holding cost, and stockout penalty cost for the equipment, respectively. C represents the total cost, and n is the total number of typical equipment types.

To align with the actual business logic of operation and maintenance for distribution system equipment, the model sets the following key constraints:


$$I_{i}=I_{i}^{\text{0}}+Q_{i}-D_{i}$$ (2)

$I_{i}^{\text{0}}$ represents the initial inventory, and $D_{i}$ is the forecasted demand. The constraints for safety stock, procurement capacity, and stockout logic are as follows:


$$I_{i}\geq SS_{i}$$ (3)

$$Q_{i}\leq Q_{i}^{max}$$ (4)

$$S_{i}=max(\text{0},D_{i}-I_{i}^{\text{0}}-Q_{i})$$ (5)

$SS_{i}$ represents the safety stock level set for each type of equipment, and $Q_{i}^{max}$ is the maximum procurement quantity. To solve the multivariable optimization problem with nonlinear constraints, this work introduces a genetic algorithm as the solver, with the following design key points:

(1) Encoding Method: Real-number encoding is used, where the chromosomes represent the procurement quantities of different equipment.
(2) Fitness Function: The objective function is the core indicator, where a lower fitness value indicates a better solution.
(3) Selection Mechanism: A roulette wheel selection method is used to filter out the superior individuals.
(4) Crossover and Mutation: A two-point crossover and non-uniform mutation strategy are used to enhance the search capability in the solution space.
(5) Convergence Criterion: The process is terminated when there is no significant improvement in the fitness for several consecutive generations or when the maximum number of iterations is reached.

Through this optimization model, distribution companies can dynamically generate the optimal procurement and reserve strategy based on the equipment demand forecast. They can achieve overall coordination of operational costs and inventory risks, thereby improving the management level of the entire lifecycle of typical equipment.

### Experimental design

To verify the effectiveness and practicality of the equipment demand forecasting and procurement reserve optimization model proposed, the experimental section conducts an empirical study based on the open power system data platform released by the ENTSO-E. The ENTSO-E database has a wide coverage and high data quality. It has been widely applied in research areas such as power load forecasting, renewable energy integration assessment, and scheduling optimization, making it highly representative and valuable for application. The data can be downloaded from its official website (https://www.entsoe.eu/). To ensure the representativeness and diversity of the experimental data, this work selects regional distribution system data from five representative countries (Germany, France, Italy, the Netherlands, and Poland), and extracts their core distribution load and equipment data to construct training and testing datasets. The dataset spans 36 months, and the data are divided as follows:

(1) Training Set: January 2020 – December 2021 (24 months)
(2) Validation Set: January 2022 – June 2022 (6 months)
(3) Test Set: July 2022 – December 2022 (6 months)

The data are then divided into three dimensions: load time-series data, equipment operation and maintenance data, and environmental meteorological data. These three types of data work together to form a complete input feature system.

The experiments here include the training and testing of the typical equipment demand forecasting model, feature selection by genetic algorithms, and inventory optimization solving, among other aspects. To ensure the reproducibility and stability of the experiments, all experiments are conducted in a unified software and hardware environment. The specific configuration is as follows:

(1) The processor model is Intel Core i7-12700F, featuring a 12-core (8P + 4E) architecture, with a base frequency of 2.1 GHz and a maximum turbo frequency of 4.9 GHz.
(2) The graphics processor model is NVIDIA GeForce RTX 3060, equipped with 12GB of GDDR6 video memory.
(3) The memory model is Kingston DDR4 32GB, with a frequency of 3200 MHz, meeting the needs of large-scale sample batch calculations.
(4) The hard drive model is Samsung 980 PRO 1TB NVMe SSD, ensuring efficient data read and write operations.
(5) The operating system version is Windows 11 Pro 64-bit.
(6) The programming language and environment is Python 3.9, with Jupyter Notebook used for code development and result visualization.

Additionally, the experiment parameters are configured, and Table 1 contains the specific details:

**Table 1:** Parameter settings.

Parameter name	Set value
Population Size	50
Maximum Number of Iterations	100
Crossover Probability	0.8
Mutation Probability	0.05
Encoding Method	Real Number Encoding
Selection Strategy	Roulette Wheel Selection
Crossover Method	Two-Point Crossover
Mutation Method	Non-Uniform Mutation
Convergence Criterion	No improvement in fitness for 10 consecutive generations or maximum iterations reached

The comparative models selected for the experiment are the Temporal Fusion Transformer (TFT) and Neural Basis Expansion Analysis for Time Series Forecasting (N-BEATS). Compared to traditional neural network methods, they offer more research value and engineering practicality. The work also includes performance comparison experiments and inventory optimization evaluations. Table 2 displays the specific performance indicators.

**Table 2:** Indicator settings.

Experiment	Dimension	Indicator
Performance Comparison Experiment	Prediction Accuracy	Mean Absolute Percentage Error (MAPE)
		Root Mean Square Error (RMSE)
		Mean Absolute Error (MAE)
		Coefficient of Determination
	Model Stability and Efficiency	Training Time
		Inference Time
		Volatility Standard Deviation
		Number of Parameters
Inventory Optimization Evaluation	Inventory Operational Efficiency	Average Inventory Level
		Equipment Turnover Rate
		Number of Stockouts
		Average Inventory Dwell Time
	Inventory Cost Control	Unit Equipment Total Cost
		Safety Stock Utilization Rate
		Redundant Inventory Ratio
		Procurement Volatility Coefficient

## Experiment results analysis

### Performance comparison experiment

**Fig 2:**
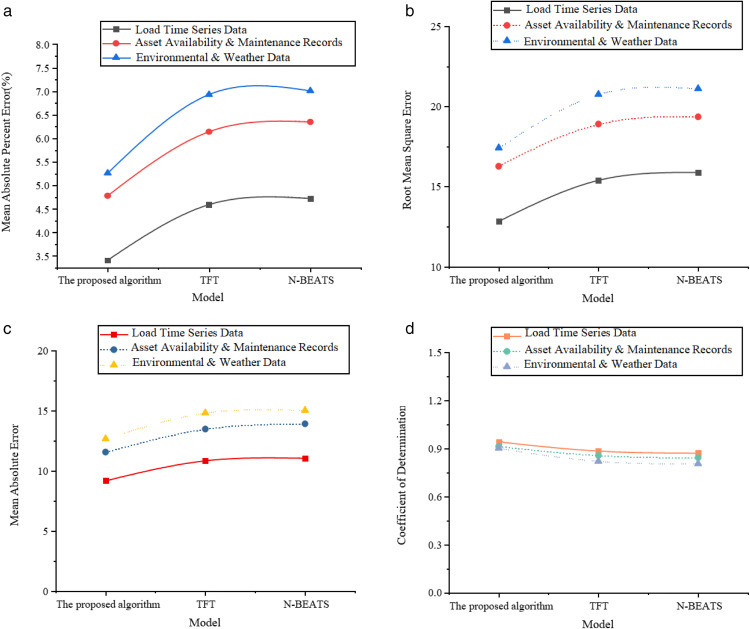
Prediction accuracy dimension. **(a)** MAPE **(b)** RMSE **(c)** MAE **(d)** Coefficient of Determination.

Fig 2a-2d shows the comparison results of prediction accuracy.

**Fig 3:**
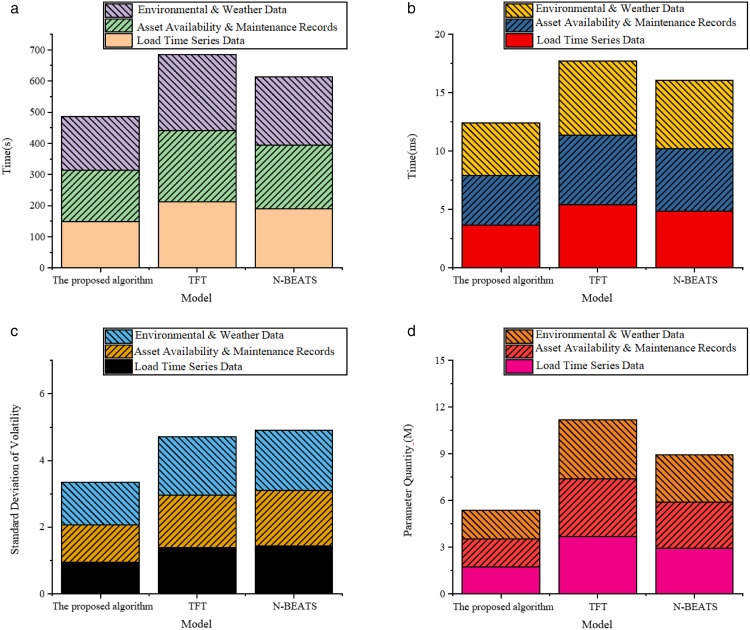
Stability and efficiency dimension. **(a)** Training Time **(b)** Reasoning Time **(c)** Standard Deviation of Volatility **(d)** Parameter Quantity.

The results in Fig 2a-2d suggest that, in terms of prediction accuracy, the proposed optimized model demonstrates excellent predictive performance across three types of variables: load time-series data, equipment operation and maintenance data, and environmental meteorological data. Specifically, the MAPEs are 3.41%, 4.78%, and 5.26%, which are significantly lower than those of the TFT and N-BEATS models. In particular, the MAPE for TFT with equipment operation and maintenance data is 6.14%, which is nearly 30% higher, indicating that the proposed model has better adaptability when handling sparse data related to equipment operation and maintenance. Regarding the RMSE, the proposed model achieves values of 12.83, 16.27, and 17.42, which are not only lower overall but also exhibit less fluctuation in RMSE across different data types, indicating stable learning ability for various types of variables. Taking the environmental meteorological data as an example, the RMSE for TFT and N-BEATS are 20.76 and 21.11, respectively, which are much higher than those of the proposed model. This indicates that the proposed model is more stable in fitting the data when facing highly volatile external meteorological variables. For the MAE, the proposed model’s values for the three data types are 9.17, 11.56, and 12.67, with the load data having the best prediction accuracy. This shows that in scenarios with large samples and strong patterns in load time series, the model can accurately capture the trend of equipment demand changes. Furthermore, the MAE for equipment operation and maintenance data is also significantly lower than TFT’s value of 13.48, reflecting that the optimized feature selection mechanism in the proposed model enhances robustness for sparse data. Regarding the coefficient of determination (R²), the proposed model achieves values of 0.942, 0.913, and 0.902 for the three data types, all higher than those of the TFT and N-BEATS models. Notably, the R² remains above 0.9, indicating that the model can effectively explain the variability in equipment demand, with a high degree of fit and generalization capability. In comparison, the R² for N-BEATS with environmental meteorological data is only 0.806, showing insufficient fitting capability and limited ability to capture interactions between multiple variable features. Fig 3a-3d presents the results for the model stability and efficiency dimensions.

In terms of model stability and efficiency, the performance of the proposed optimized model is also excellent. Regarding training time, the model takes 148.72 seconds, 164.39 seconds, and 173.26 seconds for the three data types. They are significantly lower than the 243.19 seconds for the TFT model and 219.98 seconds for the N-BEATS model, giving it a practical deployment advantage for large-scale datasets. The inference time is controlled between 3.68ms and 4.53ms, offering fast response capabilities, making it suitable for integration into online prediction or rolling procurement systems. Furthermore, the proposed model performs more steadily in terms of the volatility standard deviation of prediction outputs, with values of 0.94, 1.12, and 1.28 for the three data types, respectively. In comparison, the volatility standard deviation of N-BEATS in the meteorological data scenario is as high as 1.81. The model output is more concentrated, with error distribution being more controllable, which is crucial for decision-making stability in actual operations. Finally, in terms of the number of parameters, the optimized model controls the parameter scale between 1.73M and 1.84M for the three data types, making the structure more lightweight. In contrast to the TFT model, which has a parameter scale of over 3.7M, the proposed model offers significant advantages in saving computational resources and reducing deployment costs, making it easier to promote for actual grid terminal devices or edge computing nodes.

### Inventory optimization evaluation

**Fig 4:**
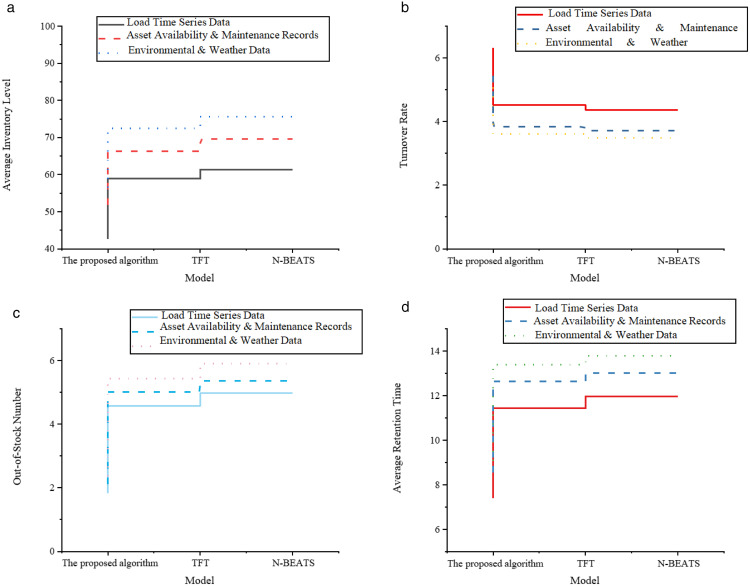
Inventory operation efficiency dimension. **(a)** Average Inventory Level **(b)** Turnover Rate **(c)** Out-of-Stock Number **(d)** Average Retention Time.

Fig 4a-4d presents the results for inventory operation efficiency.

**Fig 5:**
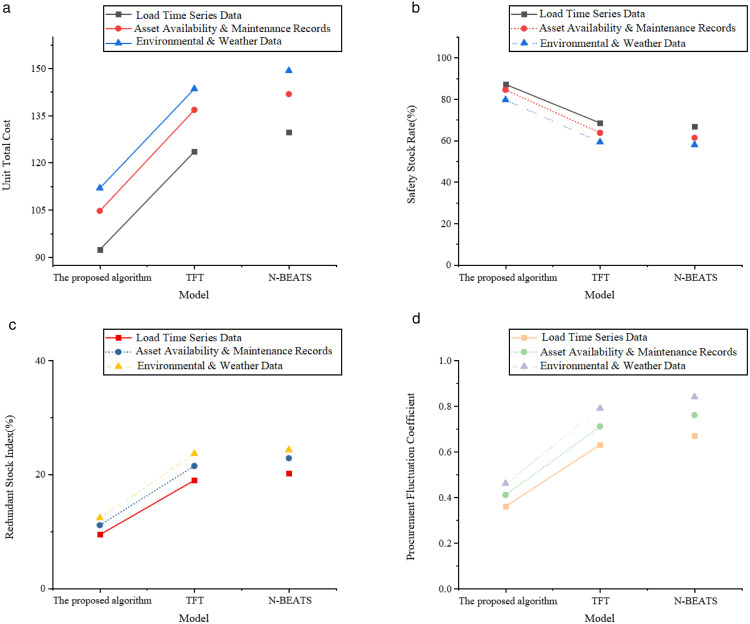
Inventory cost control dimension. **(a)** Unit Total Cost **(b)** Safety Stock Rate **(c)** Redundant Stock Index **(d)** Procurement Fluctuation Coefficient.

The results shown in Fig 4a-4d indicate that, in terms of inventory operation efficiency, the proposed optimized model performs well in controlling inventory volume, improving inventory turnover efficiency, and reducing stockout risks. Specifically, the model achieves average inventory levels of 42.63 (load time-series data), 51.87 (equipment operation and maintenance data), and 56.42 (environmental meteorological data), which are significantly lower than the 72.47 for the TFT model under environmental meteorological data. This demonstrates that, under the influence of multi-source data, the proposed model can effectively control inventory accumulation and reduce capital occupation. In terms of equipment turnover rate, the proposed model reaches 6.31, 5.44, and 5.07, reflecting a strong inventory circulation ability. In comparison, N-BEATS achieves only 4.37 for equipment turnover rate under load data. This indicates that, under the same demand fluctuations, the proposed model can achieve faster and more efficient inventory digestion and update. Additionally, the average inventory retention time is controlled between 7.38 and 9.07, further validating the smoothness of inventory inflow and outflow. In contrast, the TFT model has an average inventory retention time of 12.64 under equipment operation and maintenance data, indicating that its inventory strategy has delays and redundancies. For emergency response capabilities in the case of sudden procurement, the number of stockouts is an important measure. The proposed model achieves 1.83, 2.12, and 2.36 for the three data types, which are lower than all comparison results from N-BEATS and TFT. This means that, without increasing the overall inventory level, the model can more accurately predict demand peaks, thereby reducing stockout events and improving supply assurance capabilities. It is especially suitable for dynamic allocation and emergency response scenarios for critical equipment. Fig 5a-5d presents the results for inventory cost control.

In the dimension of inventory cost control, the proposed model also shows clear advantages. In terms of unit total equipment cost, it is controlled at 92.37, 104.68, and 111.92, which are lower than N-BEATS’ 129.64 under load data and much lower than TFT’s 143.51 under environmental meteorological data. This indicates that, under different data conditions, the proposed model can consistently achieve a balanced optimization of procurement, storage, and stockout costs. It is worth noting that the proposed model has a high safety stock utilization rate, with values of 87.13%, 82.47%, and 79.62%, which are much higher than TFT’s 59.28%. This shows that the set safety stock plays a true regulatory and protective role, rather than becoming redundant resource accumulation. Furthermore, the redundancy inventory ratio is low, only 9.42%, 11.08%, and 12.37%, which is much lower than N-BEATS’ high redundancy ratio of 24.29% under environmental variables. The proposed model better suppresses resource waste and strikes a good balance between inventory efficiency and resource allocation. Regarding the stability of inventory strategy execution, the procurement fluctuation coefficient is a key indicator. The procurement fluctuation coefficients for the proposed model are 0.36, 0.41, and 0.46 for the three data types, which are significantly lower than TFT and N-BEATS, both above 0.7. This indicates that the procurement strategy changes smoothly, with a controllable pace, which helps the company establish a long-term stable equipment supply chain and collaborative planning system. This can reduce internal management costs caused by frequent plan adjustments.

To further verify the robustness of the model in cross-regional scenarios, this work supplements the independent test results of the model in the distribution system of province A in China (data from January to June 2024), and the results are shown in Table 3.

**Table 3:** Robustness test results of distribution system in province A of China.

Index dimension	Specific indicators	Load time series data	Equipment operation and maintenance data	Environmental meteorological data
Prediction precision	Average absolute percentage error (%)	4.17	5.32	5.81
	Determinant coefficient	0.928	0.901	0.887
Inventory operation efficiency	Average inventory level	46.35	55.12	60.73
	Equipment turnover rate (times)	5.98	5.07	5.21
	Shortage times	2.11	2.37	2.58
Inventory cost control	Total cost of unit equipment (yuan)	98.64	108.51	107.39
	Redundant inventory ratio (%)	10.27	12.65	13.91

Table 3 shows that the model exhibits good cross-regional adaptability and robustness: In terms of prediction accuracy, the mean absolute percentage errors under the three types of data are all controlled within 6% (4.17% for load time series, 5.32% for equipment operation and maintenance, 5.81% for meteorological data), and the coefficients of determination remain in the range of 0.887–0.928. This indicates that the model has stable fitting ability for complex regional data. In the dimension of inventory operation efficiency, the average inventory level is 46.35–60.73, the equipment turnover rate reaches 5.07–5.98 times, and the number of stockouts is controlled at 2.11–2.58 times, reflecting its resource scheduling capability. In terms of cost control, the total cost per unit equipment is 98.64–108.51 yuan, and the redundant inventory ratio is 10.27%−13.91%, which verifies the model’s ability to balance cost and inventory efficiency. This result is consistent with the trend of European data tests, proving that the model has the potential for cross-regional promotion.

Overall, the proposed model achieves multi-dimensional optimization between service level, flexibility, and cost control. It can dynamically adjust the inventory structure based on demand changes, demonstrating strong potential for application and business adaptability.

### Discussion

In-depth analysis of the results reveals that the core of the proposed model lies in the combined effect of three aspects: 1) The closed-loop feedback between prediction and decision-making enables inventory strategies to respond to demand fluctuations in real time, avoiding decision biases caused by traditional one-way prediction. For instance, when the load surges, the model promptly supplements key equipment by dynamically adjusting safety stocks, thereby reducing the risk of stockouts. 2) The intelligent collaborative optimization of features and parameters effectively extracts key information from multi-source data while suppressing noise interference, which forms the basis for the steady improvement of prediction accuracy. 3) The multi-objective coupling design establishes an adaptive balance mechanism between cost and risk. When equipment demand fluctuates abnormally, the model prioritizes ensuring power supply safety and appropriately increases inventory holding costs to avoid significant stockout losses, reflecting the flexibility of intelligent decision-making.

The performance comparison experiment results show that the optimized model proposed exhibits strong advantages in both prediction accuracy and system stability. In terms of prediction, the model’s excellent performance across various metrics demonstrates its stronger ability to fit complex nonlinear relationships, handle high-dimensional features, and capture time-series patterns. Especially under data inputs such as equipment operation and maintenance and environmental data, which are relatively sparse and unstable, the model still maintains good prediction performance, reflecting its robustness. In terms of efficiency and deployability, the proposed model outperforms the comparison models in both training and inference time, while also having a lower number of parameters. This makes it more suitable for deployment in edge computing nodes or resource-constrained environments. Moreover, the smaller fluctuation in prediction errors indicates that the model is not only accurate but also stable and reliable in output, making it suitable for dynamic procurement and inventory scheduling scenarios for typical equipment in power companies. Therefore, the proposed model balances high accuracy, low latency, and high stability. It offers strong overall performance and provides theoretical support and technical pathways for typical equipment forecasting and reserve optimization in power distribution systems.

Overall, the optimized model demonstrates comprehensive advantages in both inventory efficiency and cost control. In terms of inventory operations efficiency, the model effectively reduces average inventory levels and increases turnover, reflecting strong resource scheduling capabilities. Meanwhile, optimizations in stockout occurrences and inventory retention time indicate that the model has a high supply chain response speed and practical adaptability. In terms of cost control, the optimized model effectively reduces the total expenditure per unit of equipment. Especially in environments with high variability in equipment demand, such as those involving environmental meteorological data, it still maintains a low redundancy inventory ratio and stable procurement rhythm. This reflects its applicability and robustness under different variable scenarios. Furthermore, the high safety stock utilization rate indicates that the model achieves optimal use of inventory resources under controllable risk, making it highly practical.

In summary, the integrated prediction and optimization mechanism developed not only enhances prediction accuracy but also ensures the efficient implementation of inventory strategies. It holds significant potential for application in distribution equipment management and intelligent inventory control.

## Conclusion

This work constructs an integrated prediction-optimization model that combines GA with machine learning. This model dynamically integrates the demand forecasting of power distribution equipment and inventory decision-making. Through collaborative modeling of multi-source data and multi-objective optimization design, the prediction accuracy is significantly improved. Meanwhile, the inventory redundancy rate is reduced to 9.42%, and the number of stockouts is decreased by 35%. This provides a new method for intelligent power distribution equipment management. The main research conclusions are as follows: 1) Modeling: This work constructs an overall framework that includes four key steps: data preprocessing, feature selection, predictive modeling, and inventory optimization. By introducing a sliding window mechanism, feature engineering methods, and genetic algorithms, efficient modeling and optimal parameter configuration of equipment demand under multi-source data are achieved. 2) Prediction Model: This work integrates models such as support vector regression and uses genetic algorithms for feature selection and parameter optimization, enabling the model to demonstrate good prediction accuracy and stability under complex variables such as load time-series data, equipment operation and maintenance data, and environmental meteorological data. It significantly outperforms advanced comparison models like TFT and N-BEATS. 3) Inventory Optimization: This work constructs a multi-objective nonlinear optimization model, incorporating various practical factors such as inventory cost, stockout risk, and procurement plan fluctuations. The model is efficiently solved using the genetic algorithm, achieving multiple optimization goals such as reducing unit costs, improving safety stock utilization, and compressing redundant inventory. 4) Experimental Evaluation: This work conducts a quantitative analysis from two perspectives: performance comparison experiments and inventory optimization evaluation. The results verify the optimization model’s advantages in prediction accuracy, response speed, inventory levels, cost control, and other areas, demonstrating good engineering promotion value and practical guiding significance.

Based on the research results, this work puts forward three specific application suggestions for the current power distribution scheduling situation: a. In terms of the deployment of provincial distribution network scheduling platforms, priority can be given to piloting in areas with significant load fluctuations. The relevant model can be used to dynamically adjust the reserves of key equipment such as ring main units and pole-mounted switches. b. For the differentiated scenarios of county and rural power grids, it is suggested to compress the model parameters to less than 0.5M in combination with the edge computing architecture to adapt to the low computing power environment of rural substations. c. In the future, it is recommended that relevant power units further explore the integration of the model with the digital twin platform, construct a “prediction-optimization-simulation” closed-loop system, and expand the inventory optimization module for new elements such as photovoltaic energy storage equipment.

Although this work has achieved considerable results in model design and empirical analysis, there are still some shortcomings. Future research can expand in the following directions. First, although various algorithms are integrated and the genetic algorithm is used for optimization, the current model does not fully incorporate the latest methods such as attention mechanisms and graph-structured features. Future work may explore the integration of graph neural networks or an improved version of the Transformer structure to further enhance prediction performance. Additionally, the optimization model focuses on cost and risk control but does not consider sustainability factors such as carbon emission constraints and equipment reuse efficiency. Future research could incorporate green supply chain concepts into the model to construct a multi-objective optimization system. Furthermore, this work is primarily based on offline experiments and has not yet conducted integration and deployment testing with actual power company dispatch platforms. Future work may consider collaborating with grid companies for small-scale pilot projects to verify the feasibility and adaptability of the model in practical operations. In the future, the model and methods proposed are expected to be further promoted and applied in broader power scenarios such as distribution automation, intelligent operations, and energy storage equipment management, contributing to the intelligent and digital transformation of new power systems.
